# Supplementary material for: Systemic LPS Administration Stimulates the Activation of Non-Neuronal Cells in an Experimental Model of Spinal Muscular Atrophy
Source: Cells. 2024 May 4;13(9):785. doi: 10.3390/cells13090785 (PMC11083572; doi:10.3390/cells13090785)
Supplement: Supplementary file 1 [file cells-13-00785-s001.zip › cells-2918326-supplementary.pdf]

Supplementary Material

Table S1. Primary antibodies used for immunohistochemistry and WB

Antibody name	Host	Catalogue number	Supplier
anti-SMN	mouse	610646	BD Transduction Laboratories
anti- beta actin	rabbit	4970S	Cell signaling
anti-GAPDH	rabbit	10494-1-AP	Proteintech
anti-Iba-1	rabbit	019-19741	Wako
anti-GFAP	rabbit	Z0334	Dako
anti – SYP	mouse	MO776	Dako
anti-CD206	goat	sc-34577	Santa cruz
anti- iNOS	mouse	sc-7271	Santa cruz
anti-S100A10	goat	AF2377	Biotechne, R & D systems
anti-complement C3b/C3c	mouse	HM1065	Hycult Bioteck

Table S2. Secondary antibodies used for immunohistochemistry and WB

Antibody name	Host	Catalogue number	Supplier
anti-mouse IgG-HRP	horse	7076S	Cell signaling
anti-rabbit IgG-HRP	goat	A0545	Sigma Aldrich
anti-rabbit IgG	goat	BA-1000	Vector
anti-rabbit 488A	goat	20012	Biotium
anti-mouse 555	goat	20030	Biotium
anti-goat 555	donkey	20039	Biotium

Sup. Material 1

ABBREVIATIONS:

A_c	Cell's area
A_p	Projection area
**AD**	Alzheimer's disease
**ALS**	Amyotrophic lateral sclerosis
**a-MNs**	Alpha Motor Neurons
**CNS**	Central nervous system
**DAB**	3,3'-diaminobenzidine
**ECL**	Enhanced chemiluminescence
**FBS**	Fetal Bovine Serum
**HRP**	Horseradish peroxidase
**IL1β**	Interleukin-1beta
**LPS**	Lipopolysaccharides
**MCP-1**	Monocyte chemoattractant protein 1 expression
**NF-κB**	Nuclear factor kappa B
**NGS**	Normal Goat Serum
**P5**	Postnatal day 5
**PBS**	Phosphate-buffered saline
**PDVF**	Polyvinylidene difluoride
**pre-mRNA**	Precursor mRNAs
**RT**	Room temperature
**SMA**	Spinal muscular atrophy
**SMN**	Survival Motor Neuron protein
**SYP**	Synaptophysin
**TLR4**	Toll-like receptor 4
**TNF-a**	Tumor necrosis factor alpha
**UBA1**	Ubiquitin Like Modifier Activating Enzyme 1
**WB**	Western blot
